# Supplementary figures and images for: DivIVA concentrates mycobacterial cell envelope assembly for initiation and stabilization of polar growth
Source: Cytoskeleton (Hoboken). 2018 Nov 30;75(12):498–507. doi: 10.1002/cm.21490 (PMC6644302; doi:10.1002/cm.21490)

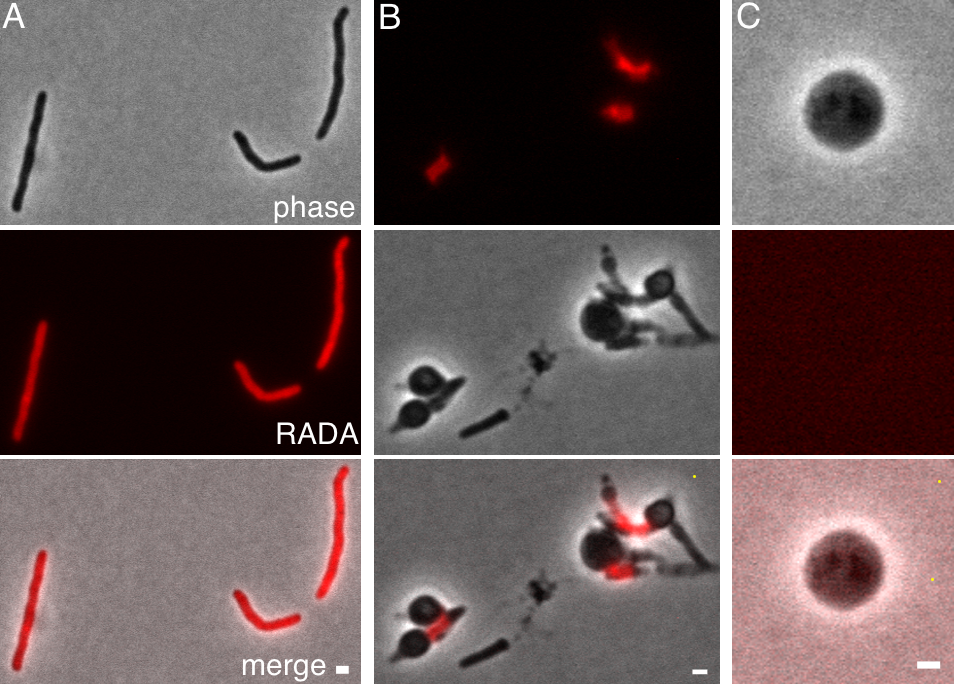

Supplement: Supplementary file 1 — FIGURE S1 Loss of peptidoglycan labeling in misshapen and spherical regions of cells during and after spheroplasting procedure. M. smegmatis were labeled overnight with RADA and imaged prior to spheroplasting, (a), after 24 hours of glycine incubation, (b), and after completion of lysozyme digestion, (c). Scale bars, 1 μm. [file CM-75-498-s001.tif]

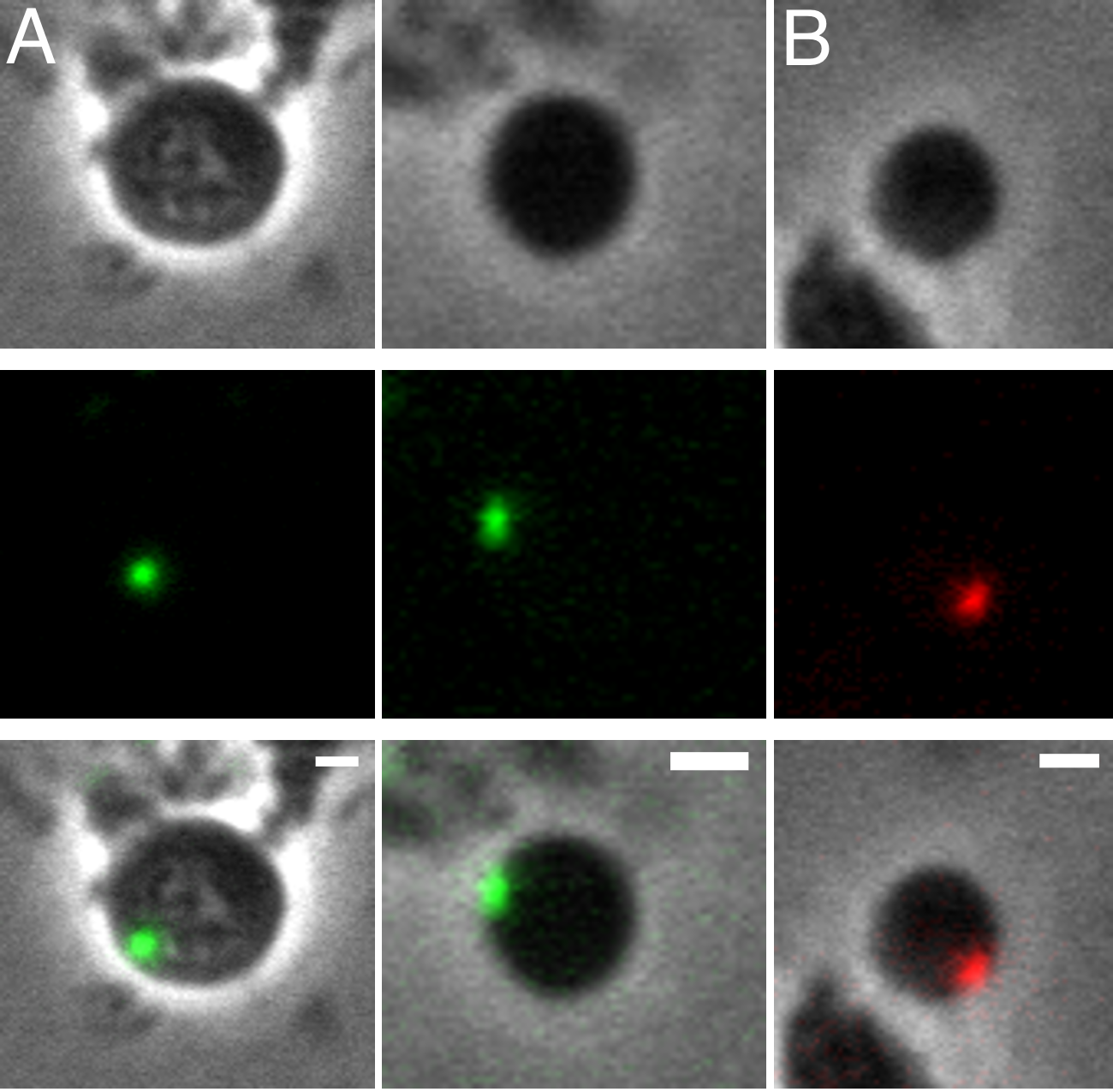

Supplement: Supplementary file 2 — FIGURE S2 DivIVA foci persist in the absence of visible negative curvature. Spheroplasts were generated from M. smegmatis expressing either DivIVA‐eGFP, (A), or RFP‐DivIVA, (B). Scale bars, 1 μm. [file CM-75-498-s002.tif]

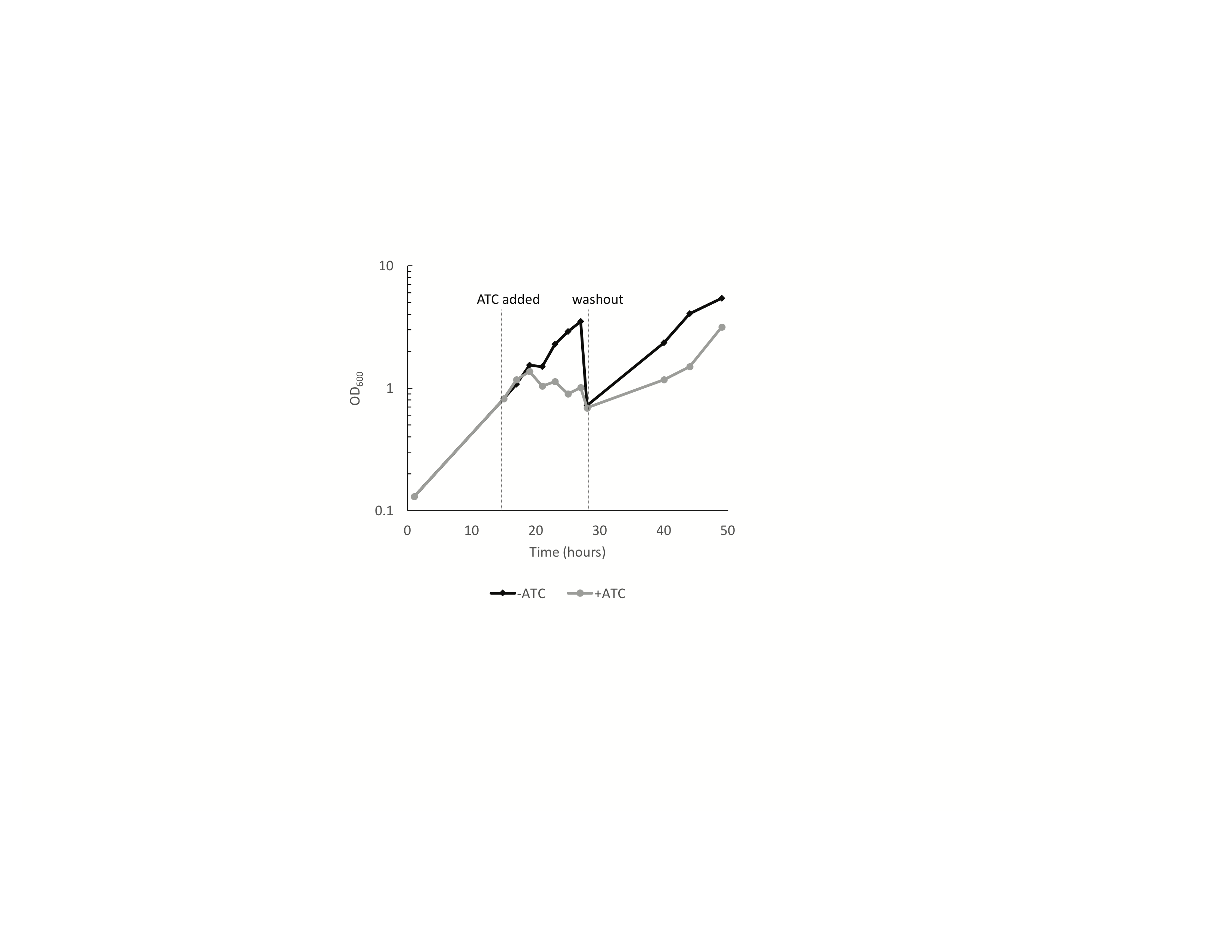

Supplement: Supplementary file 3 — FIGURE S3 Growth curves of DivIVA‐eGFP M. smegmatis during ATC‐induced depletion and following washout. DivIVA‐depleted cells grow more slowly during depletion and recovery. [file CM-75-498-s003.tiff]

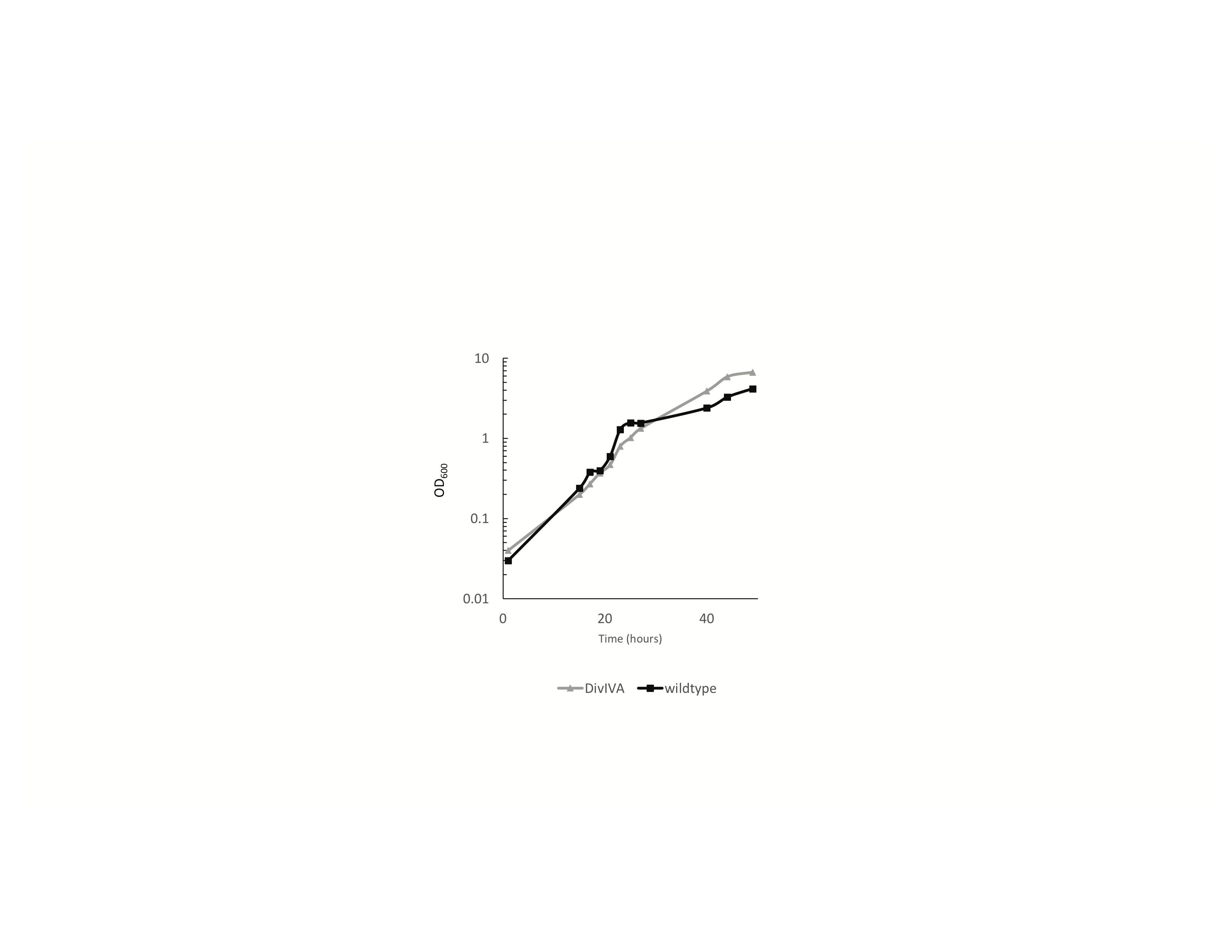

Supplement: Supplementary file 4 — FIGURE S4 Growth curves of wildtype M. smegmatis and DivVIA‐eGFP M. smegmatis. [file CM-75-498-s004.tiff]

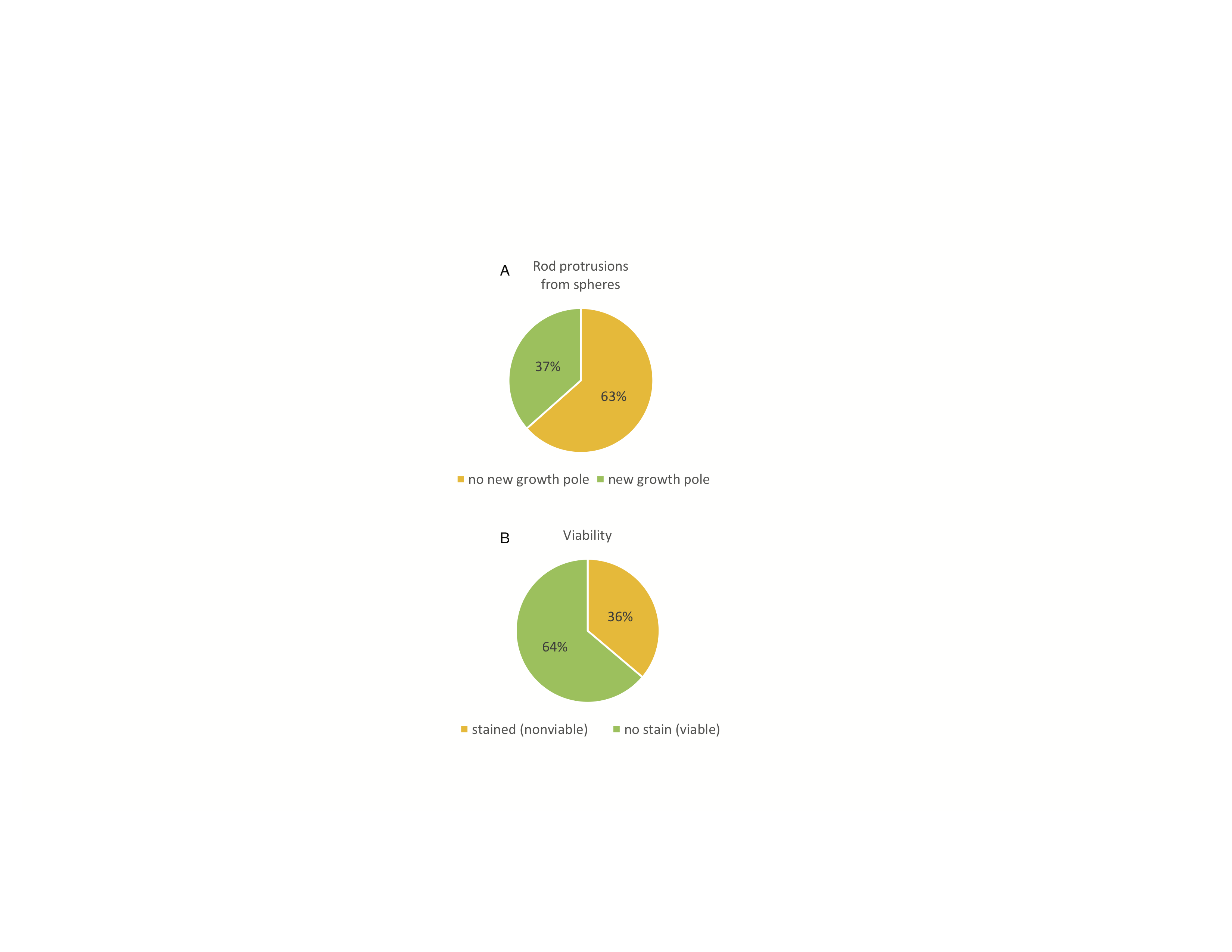

Supplement: Supplementary file 5 — FIGURE S5 Viability and rod protrusion upon DivIVA repletion. (a) Ratio of spheres that exhibited protruding growth poles after ATC washout between 10–24 hours of recovery (n = 52). (b) Ratio of DivIVA‐depleted spheres that stained viable or nonviable when treated with propidium iodide (n = 94). [file CM-75-498-s005.tiff]

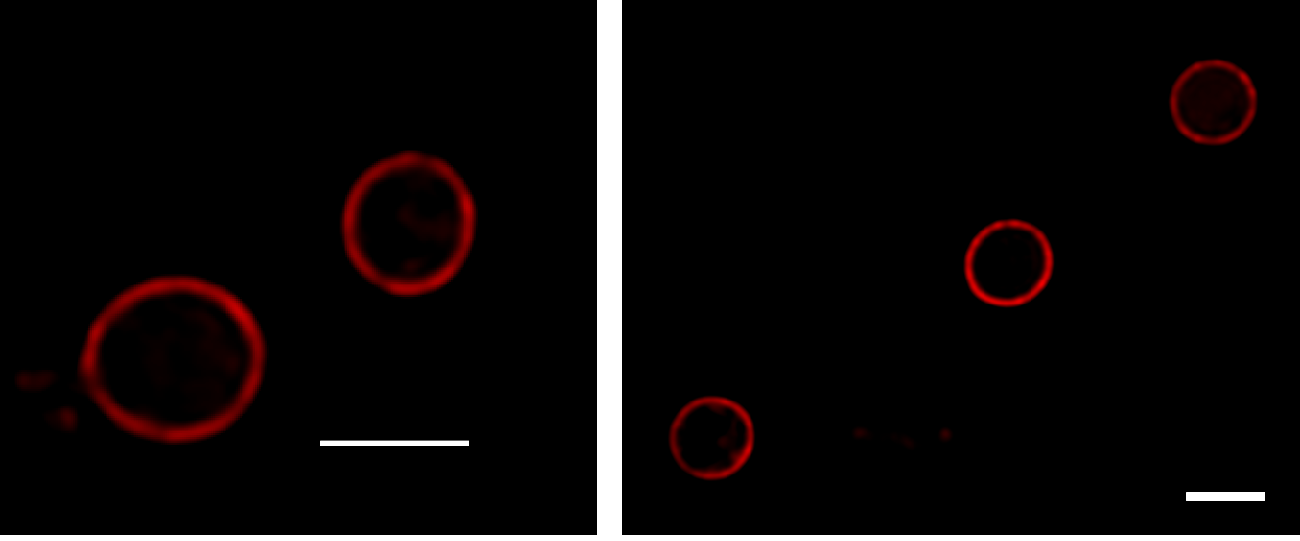

Supplement: Supplementary file 6 — FIGURE S6 alkDADA labeling of DivIVA‐depleted cells upon inhibition of periplasmic remodeling. After 12 hours of DivIVA depletion, cells were treated with 5 μg/mL clavulanate and 1 μg/mL imipenem (2X minimal inhibitory concentration) for 30 min, then labeled with alkDADA for 15 min. Labeling was detected by CuAAC with picolyl azide‐TAMRA. Scale bar, 2.5 μm. [file CM-75-498-s006.tif]
